# The development and validation of a multivariable model to predict the bleeding risk score for patients with non-valvular atrial fibrillation using direct oral anticoagulants in the Arab population

**DOI:** 10.1371/journal.pone.0250502

**Published:** 2021-05-03

**Authors:** Maha AlAmmari, Khizra Sultana, Abdulrahman Alturaiki, Abin Thomas, Monirah AlBabtain, Fakahr AlAyoubi, Hanie Richi

**Affiliations:** 1 Pharmaceutical Care Services, Ministry of National Guard Health Affairs (MNGHA), King Abdullah International Medical Research Center (KAIMRC), King Saud Bin Abdulaziz University for Health Sciences (KSAU-HS), Riyadh, Saudi Arabia; 2 Research Office, King Abdullah International Medical Research Center (KAIMRC), King Saud Bin Abdulaziz University for Health Sciences (KSAU-HS), Ministry of National Guard Health Affairs (MNGHA), Riyadh, Kingdom of Saudi Arabia; 3 Biostatistics and Bioinformatics, King Abdullah International Medical Research Center (KAIMRC), King Saud Bin Abdulaziz University for Health Sciences (KSAU-HS), Ministry of National Guard Health Affairs (MNGHA), Riyadh, Kingdom of Saudi Arabia; 4 Pharmaceutical Care Services, Prince Sultan Cardiac Center, Riyadh, Kingdom of Saudi Arabia; 5 Pharmaceutical Care Services, King Khalid University Hospital, King Saud University Riyadh, Riyadh, Kingdom of Saudi Arabia; Inselspital, Bern University Hospital, SWITZERLAND

## Abstract

**Background:**

Frequently used models, such as the HAS-BLED, ATRIA, ORBIT, and GARFIELD-AF evaluate the risk of bleeding when using an anticoagulant, for example warfarin, in patients with non-valvular atrial fibrillation. Limited studies are available reporting a model with a good discriminative ability to predict the bleeding risk score when using direct oral anticoagulants.

**Methods:**

Patient data were collected from King Abdulaziz Medical City, King Fahad Cardiac Center, and Prince Sultan Cardiac Center in Riyadh, from outpatients, inpatients, or primary care clinics. In total, 1722 patients with a prescription for a new oral anticoagulant, Dabigatran, Rivaroxaban, or Apixaban, were enrolled. A resampling approach for variable selection was used and a five-fold cross-validation to assess the model fit and misclassification probabilities. The analysis used the receiver operating characteristics curve (ROC) and the concordance (c) statistic to assess the validation models’ discriminative power. The final penalized likelihood parameters were used for the development of the risk prediction tool. The accuracy of a classification and the prediction are reported with the sensitivity, specificity, and Brier score.

**Results:**

Bleeding occurred in 11.15% of cases, of which 23.08% required a blood transfusion and 51.65% had a reduction in haemoglobin of more than 2 gm. The variable selection model identified 15 predictors associated with major bleeding. The discriminative ability of the model was good (c-statistic 0.75, p = 0.035). The Brier score of the model was 0.095. With a fixed cut-off probability value of 0.12 for the logistic regression equation, the sensitivity was 72.7%, and the specificity 66.3%.

**Conclusion:**

This model demonstrated a good performance in predicting the bleeding risk in Arab patients treated with novel oral anticoagulants. This easy to use bleeding risk score will allow the clinician to quickly classify patients according to their risk category, supporting close monitoring and follow-up for high-risk patients, without laboratory and radiological monitoring.

## Background

Non-Valvular Atrial fibrillation (NVAF) is considered the most prevalent supraventricular arrhythmia [1]. It refers to atrial fibrillation (AF) that occurs in the absence of moderate to severe mitral stenosis or mechanical prosthetic heart valves [2]. Globally, the estimated number of individuals with AF is 33.5 million [3]. The prevalence of AF increases due to several patient factors, including advanced age [4, 5], male gender [4], white race [6], comorbidities including heart failure [7], hypertension [8, 9], diabetes mellitus [10, 11], obesity [12, 13], and alcohol consumption [14]. The management of AF involves rate control, using beta-blockers or a non-dihydropyridine calcium channel antagonist, rhythm control with amiodarone, and thromboembolic prophylaxis with anticoagulant or antiplatelet therapy [15].

Compared to non-AF, AF patients are at a five-fold higher risk of developing ischemic stroke and systemic thromboembolic events [15, 16], for which they require anticoagulation. In NVAF patients, thrombus prophylaxis with anticoagulant drugs, such as unfractionated heparin [UFH] and low molecular weight heparin [LMWH], vitamin K antagonists (Warfarin), direct oral anticoagulants [DOAC] (Dabigatran, Rivaroxaban, Apixaban or Edoxaban), or antiplatelet drugs (Aspirin and Clopidogrel), must balance the embolic and bleeding risk [15]. The use of warfarin or DOAC shows a marked reduction in stroke events and all-cause mortality in NVAF patients. It decreases stroke and mortality by 60% and 25%, respectively [17, 18]. However, there are significant limitations for using warfarin. It has a narrow therapeutic window requiring frequent monitoring of the international normalized ratio (INR), a variable response due to genetic polymorphisms, dietary restrictions, and multiple drug-drug interactions [15]. Warfarin use is also associated with an increased risk of bleeding, ranging from minor bleeding to fatal intracranial or extracranial haemorrhage [18–26].

DOAC has comparable efficacy to warfarin for stroke prevention and in reducing the all-cause mortality, with lower intracranial haemorrhage in NVAF patients. In the RE-LY trial, dabigatran showed superiority to warfarin therapy for stroke reduction (RR = 0.66; 95% CI, 0.53 to 0.82; P<0.001) and non-inferiority for major bleeding (P = 0.31) [21, 22]. In the ROCKET AF trial, rivaroxaban was non-inferior to warfarin, for preventing stroke and systemic embolism (hazard ratio, 0.88; 95% CI, 0.74 to 1.03; P<0.00), and for the risk of major bleeding [23]. In addition, in the ARISTOTLE trial, apixaban was superior to warfarin in preventing stroke and systemic embolism (HR, 0.79; 95% CI, 0.66 to 0.95; P<0.001) [29], and it has a lower bleeding risk and mortality rate (HR, 0.69; 95% CI, 0.60 to 0.80; P<0.001) (HR, 0.89; 95% CI, 0.80 to 0.99; P = 0.047) compared to warfarin [24]. In the ‘ENGAGE AF-TIMI 48’ trial, Edoxaban was non-inferior to warfarin in the prevention of stroke and systemic embolism (HR, 0.87; 97.5%CI, 0.73to1.04; P = 0.08), and it was associated with significantly lower rates of bleeding (hazard ratio, 0.80; 95% CI, 0.71 to 0.91; P<0.001) and mortality [25]. However, all DOAC, with the exception of apixaban, increases the risk of gastrointestinal bleeding compared to warfarin therapy [26, 27, 29, 30].

A validated risk score supports the identification which NVAF patients are likely to benefit from anticoagulation. The widely used CHA2DS2Vasc Score (Congestive heart failure, hypertension, age 75, diabetes mellitus, stroke, vascular disease, age 65–74, sex category [female]) determines the risk for stroke [27–29]. The American College of Cardiology/American Heart Association AF guidelines recommends oral anticoagulation for a CHA2DS2Vasc score of 2 or more, as aspirin or oral anticoagulation. No therapy may be considered for a score of 1, and no therapy if the score is 0 [15]. The HAS-BLED score (Hypertension (HTN), abnormal liver/renal function, stroke, previous bleeding, labile INRs, elderly (age 65), drugs/alcohol) calculates the bleeding risk [19, 30]. A HAS-BLED score of 0 indicates low risk, 1–2 moderate risk, and 3 and more high risk [19]. A study done by Friberg et al. reported that significant bleeding rates occurred in patients with a moderate and high HAS-BLED scores of 0.7 and 2.4, with the intracranial bleeding 0.2 and 0.7 per 100 years at risk [31].

Other models, including the ORBI, Kuijer et al., Kearon et al., Shireman et al., HEMORR2HAGES, RIETE, HAS-BLED, ORBIT, and ATRIA have also been used for warfarin to calculate the bleeding risk score [30, 32–39]. In Saudi Arabia, several studies were conducted with AF patients [40, 41]. They reported that the most prevalent comorbidity reported in literature was hypertension, ranging from 59% to 80%. One study assessed the risk of bleeding with the HAS-BLED score and indicated that 63% of the patients prescribed warfarin due to AF, had a moderate risk of bleeding, 27.7% a high risk, and 9.1% a low risk, and the major bleeding rate was 6% [42]. Based on the available evidence, limited studies report a bleeding risk prediction score for the DOAC with a good discriminative model ability [42]. This cohort study aimed to develop and validate a new model for the bleeding risk prediction score in patients using DOACs due to NVAF in the Arab population.

## Methodology

**Fig 1:**
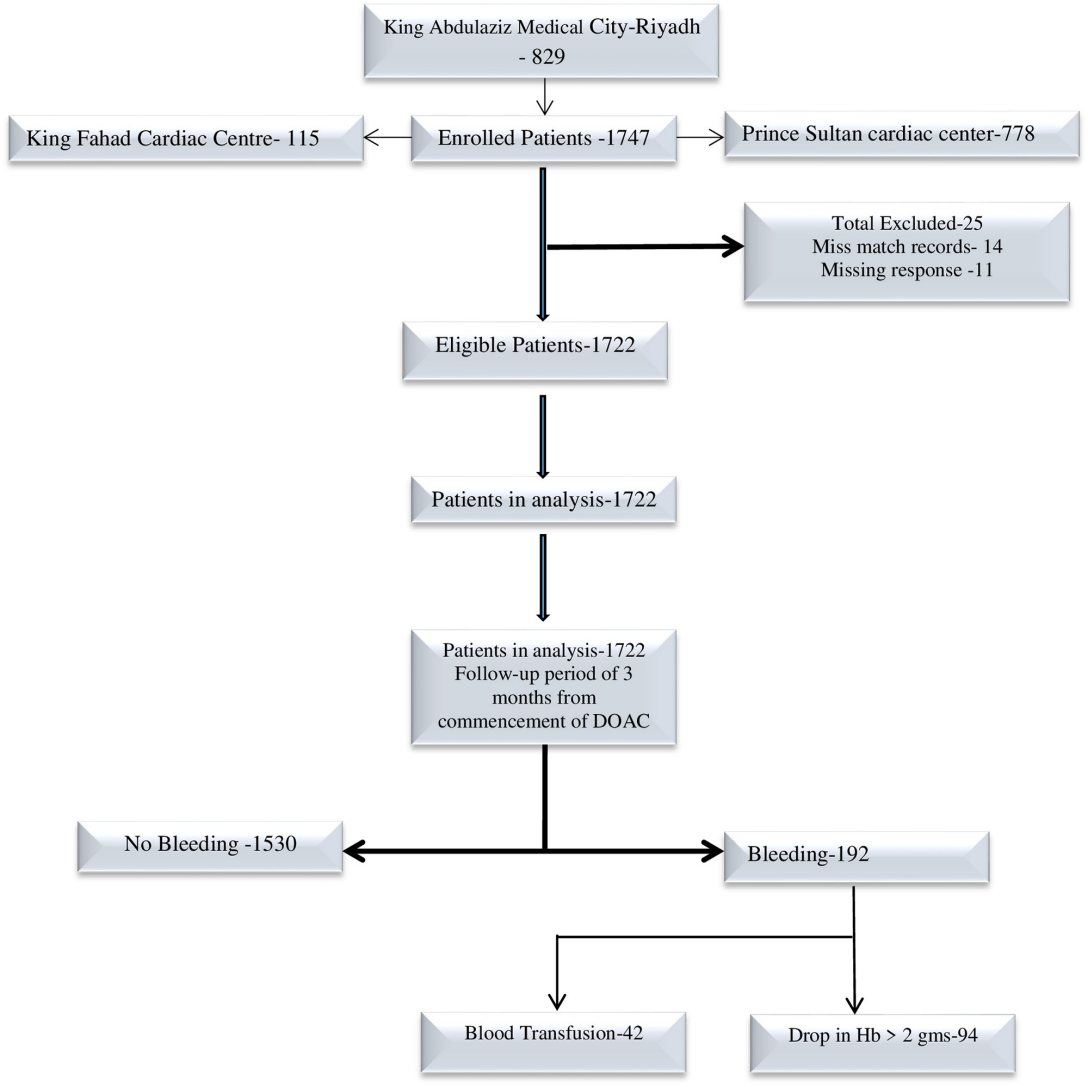
Derivation of study population.

This retrospective cohort study included 1722 patients from three centers in Riyadh, 829 patients from King Abdulaziz Medical City (KAMC), 115 patients from King Fahad Cardiac Center (KFCC), and 778 patients from Prince Sultan Cardiac Center (PSCC). KAMC has more than 1500 beds, and serves National Guard employees and their families. PSCC has 174 beds serving armed forces personnel and their dependents and other eligible patients. KFCC has 1200 beds serving its employees and the Saudi population. All three centers are government-funded multi-specialty hospitals that provide primary to tertiary healthcare. The study included eligible patients who visited these centers as an outpatient, inpatient, or primary care clinic patient from January to December 2016. The data were collected retrospectively from electronic patient records (Hospital Information system) by trained research coordinators. Fig 1 describes the derivation of the sample.

### Participants

We selected male and female patients, 18 years and older. The patients were diagnosed with NVAF (Persistent or Paroxysmal) and were using any of the listed DOAC (Dabigatran, Rivaroxaban, or Apixaban) for a minimum of one year. Edoxaban is not available in our hospital and excluded from the study. The patients had one or more of the following comorbid conditions: cardiovascular disease (hypertension (HTN) or heart failure), diastolic, systolic blood pressure, coronary artery disease (CAD), diabetes mellitus (DM), malignancy (specifically gastrointestinal (GI)), stroke, or transient ischemic attack (TIA), thyroid disorder, liver disease (hepatitis, cirrhosis, hepatic cell carcinoma, other liver diseases).

#### Primary outcome and bleeding

The primary outcome was a bleeding event or clinically relevant non-major bleeding (CRNM). Major bleeding was defined according to the guidelines of the International Society of Thrombosis and Hemostasis (ISTH) criteria as a fatal bleeding, a history of blood transfusion within 3 months from starting DOAC, a history of a reduction in the hemoglobin level of more than 2gm within 3 months from beginning DOAC, or symptomatic bleeding in a critical organ area, including intracranial, intraocular, intraspinal, retroperitoneal, intra-articular, pericardial or intramuscular with accompanying compartment syndrome [43]. A CRNM was defined according to the ISTH classification as a bleeding event that requires medical intervention by a healthcare professional or leads to hospitalization or a face-to-face evaluation as it does not fit the definition of major bleeding. Major bleeding and CRNM were analyzed together [43].

#### Predictors of bleeding

The predictors of bleeding were 1. Age, 2. Concomitant drug (Aspirin, Clopidogrel & other antiplatelets), 3. Uncontrolled blood pressure (above 150 systolic). 4. Liver failure (liver enzymes AST or ALT greater than 2 times the upper standard limit or greater than 3 times the standard upper limit), 5. Renal failure (according to the KADIGO guidelines as CKD1, CKD2, CKD3, CKD4, CKD5 or dialysis), 6. Gender: male and female, 7. Comorbidities: cardiac disease (HTN, diastolic, systolic, CAD, other cardiac diseases), 8. Liver disease (hepatitis, cirrhosis, hepatic cell carcinoma, other liver disease), 9. AST: normal and abnormal, 10. ALT: normal and abnormal, 11. CNS disease: stroke, TIA, other CNS, 12. AF: paroxysmal, persistent, others.

#### Sample size

A pilot sample of 44 patients was randomly selected from the study population. A prevalence rate of 11.3%, confidence limit 95% (4.0%, 23%) was observed for bleeding, and incomplete or missing data were observed in less than 3% of the participants. A total of 15 variables, 14 categorical and one continuous variable, were considered for the model development. Allocating at least five events for each class of variables creates a requirement of 145 events in the sample. Assuming 11.3% prevalence and 3% incomplete or missing response, a minimum sample size of 1302 participants was calculated. Considering the additional requirement of samples for model validation and the variability in the estimated prevalence from a pilot study, we estimated the sample size to be 1700 participants.

#### Ethics

Ethical approval was obtained from the Institutional Review Board of King Abdullah International Medical Research Center (KAIMRC) with approval no. RC17/139/R. The study protocol conforms to the guidelines of the Declaration of Helsinki.

#### Analysis

We use MS Excel (Microsoft Excel 2010) to create the data collection template for data collection. The investigators shared the completed template with the data collection team in the different centers. The flow of patients for the analysis is depicted in Fig 1. The data underwent rigorous six eye quality checks and was imported into SAS software (SAS Software 9.4, Copyright (c) 2002–2012 by SAS Institute Inc., Cary, NC, USA.) for data analysis and model preparation. The analysis summarized the categorical variables as frequency and percentage and the mean and standard deviation to describe age.

Follow-up started from the commencement of DOAC to 3 months afterwards to check for a major bleeding event from January to December 2016. The overall proportion and corresponding 95% confidence limit for the bleeding event and its complications were estimated. In addition to the descriptive analysis, all the variables were compared in terms of the bleeding event status, using a chi-square (or Fisher exact) test and an independent-sample ’t’ test (or Wilcoxon-test). A p-value less than 0.05 was considered significant.

#### Variable selection for model building

The analysis used a resampling approach for variable selection. The model fitted the logistic regression using the backward elimination method in randomly sampled data. The process was repeated 1000 times with different samples. The variables selected at least 100 (10%) times were included in the final model. After the variable selection, we used a 5-fold cross-validation to assess the model fit and misclassification probabilities. Logistic regression using a penalized maximum likelihood estimation method (Firth penalty) was used to fit the cross-validation model. The concordance (c) statistic and receiver operator characteristics (ROC) curves were used for assessing the validation models’ discriminative power. The value of the c statistic at 0.50 indicated no discrimination, 0.51–0.69 poor, 0.70–0.79 acceptable, 0.80–0.89 excellent, 0.90–0.99 outstanding, and 1.00 perfect discrimination [44].

We use the penalized likelihood parameters for the development of the risk prediction tool. The accuracy of a classification and the prediction are reported using sensitivity, specificity, and Brier score. Finally, we developed an MS Excel calculator using the logistic regression equation. The tool estimates the likelihood of bleeding for individual patients after entering their characteristics.

## Results

After DOAK use, 11.15% (9.75%–12.72%) bleeding events were reported. Of this group, 23.08% (17.55%–29.72%) reported a history of blood transfusion and 51.65% (44.43%–58.80%), a reduction of more than 2gm in the Hb level. Data was not clear for 10 patients whether there was a drop in hemoglobin or a blood transfusion, thus it is reported as missing information regarding severe bleeding. (Table 1).

**Table 1:** Proportion of bleeding cases.

	Yes (%)	Total(n)
Bleeding Event	192 (11.15)	1722
Blood Transfusion	42 (23.08)	182
Reduced HB more than 2g	94 (51.65)	182
Missing information about severe bleeding	10	192

### Distribution of covariates by bleeding versus no-bleeding

Table 2 summarizes the distribution of the variables for the bleeding and non-bleeding groups. An independent sample t-test with an unequal variance assumption was used for estimating the P-value for age. For the categorical variables, a chi-square test was used to generate P-values. The demographic variable associated with bleeding was the mean age of 70.01±11.15 SD and the male gender (51.57%). Concerning comorbidities, 95% of the bleeding group had cardiac disease with the highest probability of having HTN (82%), followed by CAD (37%). There was a 9.4% probability of liver disease in the bleeding group, including 4.7% cirrhosis and hepatitis. In addition, CKD2 and CKD3 renal disease had an almost equal prevalence of 38%. There was a 25% chance of bleeding in patients with a CNS comorbidity, stroke was the highest (18%). The bleeding group had a 20% chance of thyroid disease and 48% for other comorbidities. In this group, 60% had paroxysmal AF compared to 29% with persistent AF, 37% used aspirin, higher than Clopidogrel use (15%). The majority of the bleeding group used Rivaroxaban (69%), followed by Apixaban (27%) and Dabigatran (4%).

**Table 2:** Proportions of bleeding and non-bleeding events distributed by covariates.

Variable		No Bleeding	Bleeding	P-value
		Mean (StDev)	Mean (StDev)	
Age		67.50 (13.57)	70.01 (11.15)	0.0045
	**Category**	**Frequency (%)**	**Frequency (%)**	
Gender				0.5870
	Male	789 (51.57)	103 (53.65)	
	Female	741 (48.43)	89 (46.35)	
**Comorbidities**		1472 (96.21)	188 (97.92)	0.2313
Cardiac disease	Yes	1402 (91.63)	182 (94.79)	0.1288
	HTN (Yes)	1203 (78.68)	157 (81.77)	0.3213
	Diastolic HF (Yes)	360 (23.58)	43 (22.4)	0.7161
	Systolic HF (Yes)	232 (15.19)	28 (14.58)	0.8241
	CAD (Yes)	376 (24.67)	71 (36.98)	0.0003
	Other cardiac disease (Yes)	428 (27.97)	50 (26.04)	0.5731
Liver disease	(Yes)	63 (4.12)	18 (9.38)	0.0012
	Hepatitis (Yes)	20 (1.31)	9 (4.69)	0.0030*
	Cirrhosis (Yes)	29 (1.9)	9 (4.69)	0.0309*
	Hepatic cell carcinoma (Yes)	8 (0.52)	1 (0.52)	1*
	Other liver disease (Yes)	30 (1.96)	10 (5.21)	0.0099*
AST				0.4391*
	Normal	971 (94.73)	75 (92.59)	
	Abnormal	54 (5.27)	6 (7.41)	
ALT				0.6471
	Normal	1279 (96.97)	163 (97.6)	
	Abnormal	40 (3.03)	4 (2.4)	
Renal Disease				< .0001
	None	219 (15.58)	4 (2.19)	
	CDK 1	308 (21.91)	31 (16.94)	
	CDK 2	485 (34.5)	70 (38.25)	
	CDK 3	344 (24.47)	71 (38.8)	
	CDK 4 or above	50 (3.56)	7 (3.83)	
CNS disease	(Yes)	249 (16.27)	48 (25)	0.0026
	Stroke (Yes)	163 (10.66)	34 (17.71)	0.0038
	TIA (Yes)	39 (2.55)	7 (3.65)	0.3752
	Others CNS (Yes)	74 (4.84)	12 (6.25)	0.3967
Uncontrolled BP	(Yes)	195 (13.84)	28 (14.97)	0.6744
DM	(Yes)	913 (59.71)	112 (58.33)	0.7137
Malignancy GI	(Yes)	19 (1.24)	2 (1.04)	1*
Thyroid disorder	(Yes)	218 (14.29)	38 (19.79)	0.0435
Other comorbidities	(Yes)	608 (39.74)	93 (48.44)	0.0207
AF characteristics				0.0221
	Others	173 (14.37)	20 (11.11)	
	Paroxysmal	789 (65.53)	108 (60)	
	Persistent	242 (20.1)	52 (28.89)	
Other AF	(Yes)	234 (15.29)	27 (14.06)	0.6537
Aspirin		444 (29.02)	71 (36.98)	0.0232
Clopidogrel		159 (10.39)	29 (15.1)	0.0484
Other antiplatelet		4 (0.26)	1 (0.52)	0.4470*
DOAK follow-up				< .0001
	Dabigatran	132 (8.63)	7 (3.65)	
	Rivaroxaban	597 (39.02)	133 (69.27)	
	Apixaban	801 (52.35)	52 (27.08)	
History of other DOAK use		231 (15.1)	20 (10.42)	0.0831

* Fisher exact test is used to generate the P-value.

### Predictor variable selection and model building

The variables AST, ALT, and AF were removed from the model fitting due to a high frequency of missing values (more than 170 (10%) participants) (Table 3). We adopted the complete-case analysis approach. The variable selection process included all other variables. The resampling methodology described in the Methods section was used to iterate the final list of variables for modelling. The selected variables included age, DOAK being followed-up, history of other DOAK use, renal disease, liver disease, cirrhosis, hepatitis, CNS disease, stroke, cardiac disease, CAD, other-comorbidities, antiplatelet use, and aspirin. The gender variable was independently added to the model. We conducted a five-fold cross-validation to evaluate the performance of the model. We used the ROC curve and the AUC to report the predictive capability of the model. The results indicate that the model is very stable, and the average AUC or ’c’ statistic of the validation is 0.749 (0.035). Table 4 indicates the AUC estimates of 5 training and validation datasets. Due to the penalized likelihood estimation method, the corresponding odds ratios will be biased and not reported in the manuscript. The model calculates the bleeding events in the first 3 months after starting DOAC. Fig 3 provides a snapshot of the Excel tool. The estimated likelihood of bleeding changes with the values selected in Excel from the dropdown menu. We provided this excel in the additional files (S1 File).

**Table 3:** Missing values.

Variable	Missing values
AST	616 (35.8%)
AF characteristics	338 (19.6%)
ALT	236 (13.7%)
Uncontrolled BP before DOAK	126 (7.3%)
CAD	6 (0.3%)
Thyroid disorder	4 (0.2%)
Diastolic HF	3 (0.2%)
Systolic HF	3 (0.2%)
Cirrhosis	3 (0.2%)
Hepatitis liver disease	2 (0.1%)
Hepatic cell carcinoma	2 (0.1%)
Malignancy (GI)	2 (0.1%)
DM comorbidities	1 (0.1%)
HTN cardiac disease	1 (0.1%)
Stroke CNS	1 (0.1%)
TIA	1 (0.1%)

**Table 4:** The AUC estimates for 5-fold cross-validation.

ID Number	Training Data	Validation Data
1	0.791	0.702
2	0.778	0.723
3	0.763	0.756
4	0.764	0.784
5	0.772	0.777

**Fig 2:**
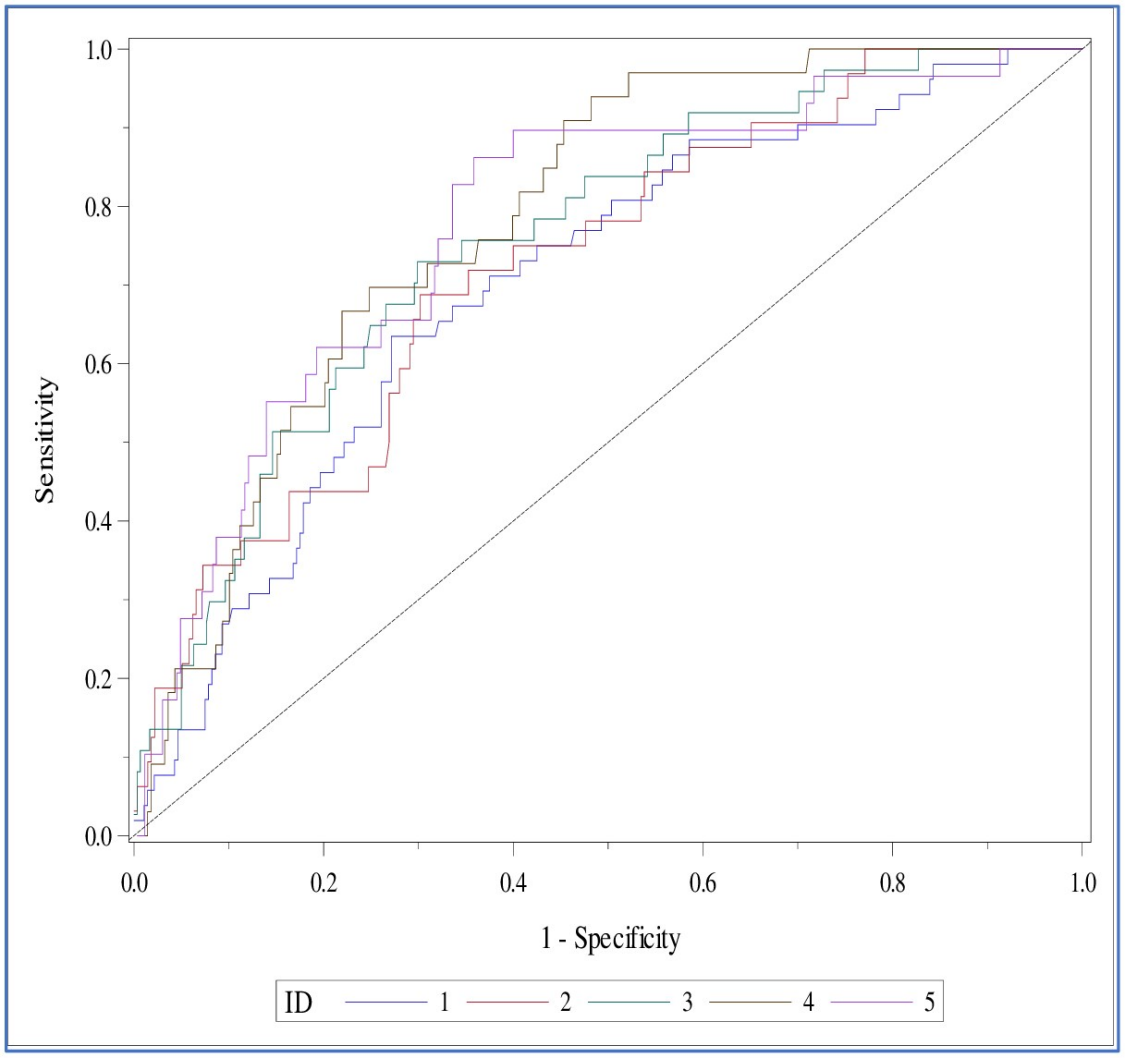
The ROC curves for the validation datasets for the five-fold cross-validation.

Fig 2 shows the ROC curves for the model validation. The curves have very slight variations and represent stable discrimination.

**Fig 3:**
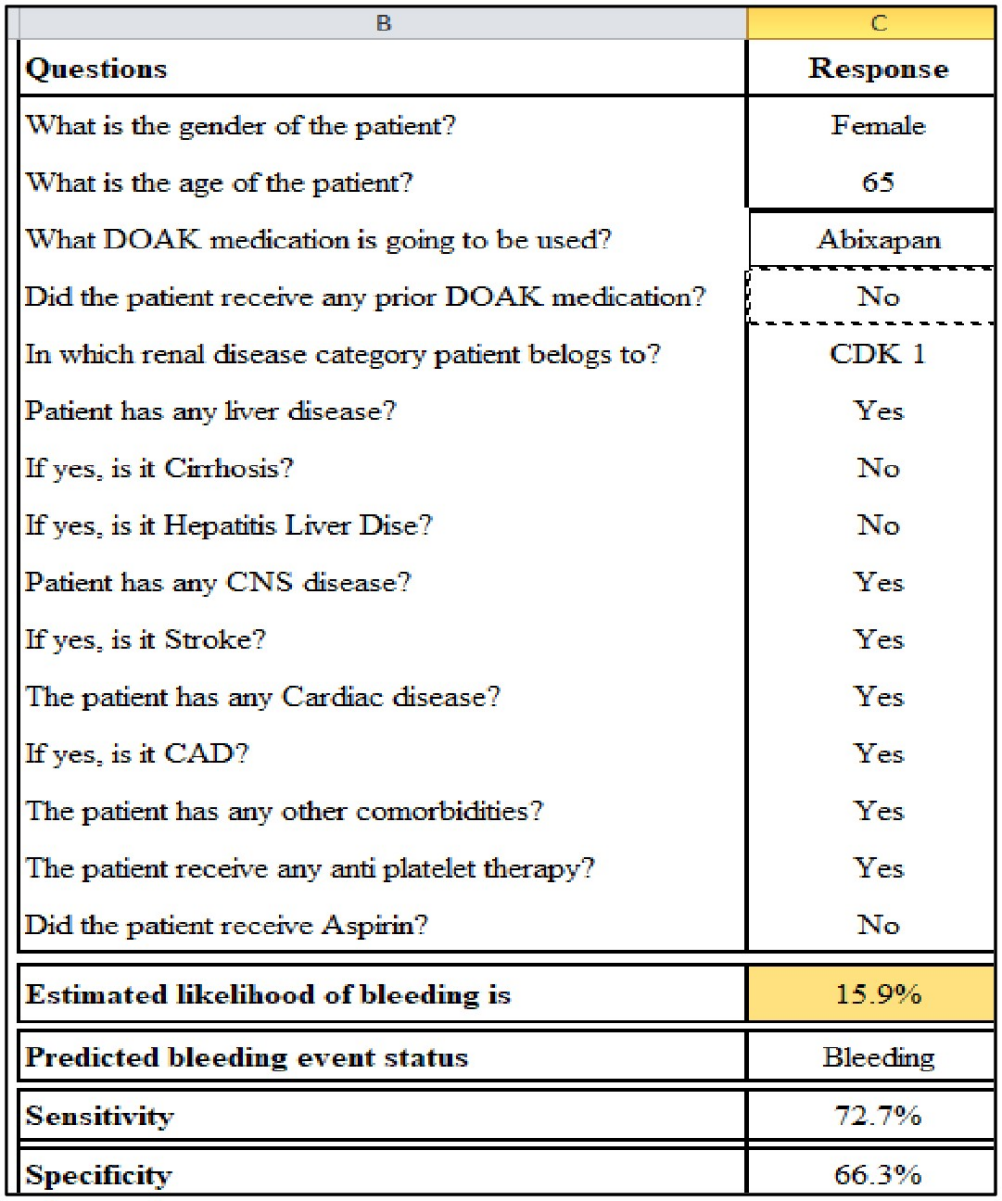
GAMA_RL_ STC score calculator.

The AUC or the c-statistic value in both the training and validation data sets was greater than 0.7 (Table 4). The Brier score estimate for the model’s predictive accuracy is 0.095, and the c-statistic was 0.75. We fixed the cut-off probability value at 0.12 for the logistic regression equation, with 72.7% sensitivity and 66.3% specificity. Table 5 contains the parameter estimates for the final model with the corresponding standard error and P-values. Fig 3 displays the parameters included in the MS Excel to obtain the predictive score (S1 File).

**Table 5:** Parameter estimates, standard error, and P-values of the final model.

Parameter (Category)	Estimate (SE)	Pr > ChiSq
Intercept	-1.8605 (0.6159)	0.0025
Gender (Female)	-0.0274 (0.0879)	0.7553
Age Demographics	0.0134 (0.00701)	0.0554
LastDOAK (Dabgatran)	0.0791 (0.2855)	0.7819
LastDOAK (Rivaroxiban)	0.933 (0.1794)	**< .0001**
Other Comorbidities (Yes)	0.3012 (0.0935)	**0.0013**
PriorDOAK (Yes)	0.3868 (0.1448)	**0.0076**
Renal Disease (CDK 1)	0.169 (0.2113)	0.4237
Renal Disease (CDK 2)	0.2801 (0.176)	0.1115
Renal Disease (CDK 3)	0.3928 (0.1832)	**0.0321**
Renal Disease (CDK 4 or above)	0.3992 (0.3683)	0.2784
Aspirin (Yes)	0.0884 (0.1898)	0.6414
Liver Disease (Yes)	0.3317 (0.269)	0.2175
Cirrhosis (Yes)	0.4358 (0.3025)	0.1497
CNS Disease (Yes)	0.1058 (0.1621)	0.5141
Stroke CNS (Yes)	0.1599 (0.1868)	0.392
Hepatitis (Yes)	0.2726 (0.2997)	0.3632
CAD (Yes)	0.1274 (0.0973)	0.1905
Antiplatelet drug (Yes)	0.1035 (0.1913)	0.5885
Cardiac Disease (Yes)	0.2482 (0.1933)	0.199

The Excel score (S1 File) calculator uses a simple user interface to enter the patient data and produce an instant risk score. A green colour code indicates safe (cut-off 12%-no bleeding), yellow a moderate or borderline risk (cut-off ≥12%≤50%), and red high risk (cut-off ≥51%).

## Discussion

This study aimed to develop a new validated model, the GAMA_RL_STC score (Gender, Age, Morbidity, Abnormal-Renal or Liver function, Stroke, and CAD) to predict the risk of bleeding in a group of patients with NVAF and treated with DOACs. We derived the bleeding risk score using a logistic regression model with 15 predictors.

To date, nine scores have been proposed to evaluate the bleeding risk in patients on oral anticoagulants such as warfarin [39]. These are ORBI, Kuijer et al., Kearon et al., Shireman et al., HEMORR2HAGES, RIETE, HAS-BLED, ORBIT, and ATRIA [30, 32–39]. All categorize patients in three categories (low, intermediate, and high-risk) and are limited due to a retrospective study design using hospitalized patients, except the HAS-BLED, which also included data from ambulatory patients [27, 31, 45]. They require demographic, clinical, and laboratory data to calculate the bleeding risk. Certain variables such as peptic ulcer disease, genetic factors, and drug abuse were included in the models proposed by Kearon et al., HEMORR2HAGES, and Shireman et al. However, the data for these risk factors was not available in their studies [33–35]. The models main outcome variable was major bleeding and clinically relevant non-major bleeding within one year of follow-up. The models were not built to predict the clinically relevant non-major bleeding and non-major bleeding. For all these models, the ’c’ statistic to predict major bleeding was less than 0.7, except for Shireman et al. and ORBIT, which had acceptable performance [35, 36, 45]. For the clinically relevant non-major bleeding and non-major bleeding, the ’c’ statistic was reported as 0.407 to 0.559 and 0.438 to 0.582, respectively, which indicated poor model performance [39]. Some of the models, including Kearon et al. and RIETE did not report the ’c’ statistic [32, 33]. It was also reported that the models misclassified the patient who had major bleeding events in other studies [39]. Most of the scores have been developed using randomized trials or registries instead of real-life cohorts [46].

Based on the evidence present in literature for these models, the international guidelines recommend the HAS-BLED score for use, as it has been validated in an European and American cohort [2, 15]. Due to the popularity of the HAS-BLED score, it has been used to assess the bleeding risk with using DOAC. A retrospective case-control study by Gorman et al. indicates that using the HAS-BLED assessment tool in patients receiving rivaroxaban demonstrated some diagnostic ability to predict major bleeding events, which was not statistically significant due to a limited sample size (c statistic = 0.68; p = 0.07) [47]. Another study that used the HAS-BLED for 88 patients on dabigatran, reported a sensitivity of 92.9% and specificity of 81.1% but did not report the discriminative ability of the model (‘c’ statistic) [48].

There is a lack of a validated scoring system for DOAC to assess bleeding events in literature [49]. Recently, two studies proposed a model for assessing the bleeding risk in patients with AF using DOACs in the Norwegian population (ABS Score) and the US population (ABH score) [46, 50]. These studies investigated the hazard rates and individual predictors of time to the bleeding event using cox proportionality hazard models. In contrast, our study focused on predicting an event’s likelihood using a penalized logistic regression method. All three studies infer that any abnormality related to the vital organs such as the heart, kidney, and liver promotes bleeding in patients. Even with a smaller sample size, our study reports higher accuracy estimates (c-statistics 0.75) for the model compared to the Norwegian (c-statistic 0.68) and US (c-statistic 0.68) studies. To date, none of the models has been validated in the Arab population. We did not validate the HAS-BLED score in the Arab population. It was observed that many patients had missing data for aspartate aminotransferase, alanine aminotransferase, alkaline phosphatase and INR in our population. Additionally, almost all patients do not consume alcohol in Saudi Arabia. We wanted to develop a model that do not require laboratory and labile INR monitoring and alcohol use. According to a study, the Norwegian ABS model calculating the bleeding risk score for DOAC, showed a higher discrimination ability than the existing models. (HEMORR2HAGES, HAS-BLED, ATRIA, and ORBIT) [50].

## Limitations and strengths

The GAMA_RL_ STC score is the first proposed model to predict the bleeding risk in patients prescribed DOAC, validated in the Arab population. It is a very user-friendly model. We use the simple penalized logistic method for predicting the bleeding event. The equation was converted to a score calculator using MS Excel (S1 Appendix). The Excel score calculator uses a simple user interface to enter the patient data and produce an instant risk score. A green color code indicates safe, yellow moderate or borderline risk, and red a high risk. Fifteen variables were considered and scored according to the binary scale to predict bleeding risk. This score applies to people who do not consume alcohol. It also does not require genetic variables and laboratory data. We used a cohort study design to derive our population. This model provides the basis for use all over KSA and in the Arab world. This model does not include the liable INR profiles of the patients. It also cannot predict clinically relevant non-major bleeding and non-major bleeding events. The follow-up of our patients was beyond the scope of the study. Additional research is required to compare our model with the existing models proposed for bleeding risk prediction for DOAC patients to evaluate its performance. External validation of our model can be done in other populations.

## Conclusion

In this cohort study of Saudi patients with AF who are prescribed DOACs, we identified strong predictors for bleeding. This easy to use bleeding risk score will allow the clinician to quickly classify patients according to their risk category and closely monitor and follow-up high-risk patients without laboratory and radiological monitoring.

## Supporting information

S1 File Calculates the bleeding risk prediction score. Excel based calculator that predicts the three months bleeding risk score.
(XLSX)

S1 Appendix (XLSX)
